# Examining Users’ Acceptance Intention of Health Applications Based on the Technology Acceptance Model

**DOI:** 10.3390/healthcare13060596

**Published:** 2025-03-09

**Authors:** Jae Hyung Park, Chul Won Lee, Chanwook Do

**Affiliations:** 1 Department of Sport Industry Studies, College of Educational Sciences, Yonsei University, Seoul 03722, Republic of Korea; pjayhsports@gmail.com (J.H.P.); wakeford@yonsei.ac.kr (C.W.L.); 2 Department of Kinesiology & Sport Management, College of Education & Human Development, Texas A&M University, College Station, TX 77843, USA

**Keywords:** technology acceptance model (TAM), mobile health application, customer satisfaction, behavioral intention

## Abstract

**Background/Objectives:** Mobile health applications are essential for improving healthcare access and promoting healthy lifestyles. This study investigates the roles of perceived ease of use, usefulness, and satisfaction in shaping behavioral intentions using the Technology Acceptance Model. **Methods:** A survey of 329 mobile health application users in Korea was analyzed using confirmatory factor analysis and structural equation modeling to test hypothesized relationships. **Results:** The findings indicate that perceived ease of use positively influences perceived usefulness (H1). Customer satisfaction is positively influenced by both the perceived ease of use and perceived usefulness (H2 and H3). Furthermore, behavioral intention is affected by perceived ease of use, perceived usefulness, and customer satisfaction (H4 through H6). **Conclusions:** The findings from this study elucidate how mobile health applications can enhance users’ perceptions of ease of use and usefulness, thereby influencing their behavioral intentions through increased satisfaction. This research also advances our understanding of application services and informs the development of effective operational and marketing strategies.

## 1. Introduction

Technological advancements have significantly transformed the contemporary healthcare industry [1]. Particularly, the widespread adoption of electronic devices (e.g., smartphones and smart watches) has encouraged many individuals to take a greater interest in their health [2,3]. Through these smart devices, individuals can monitor and manage their health information in real-time while also personalizing the prescription and treatment of various diseases based on their specific circumstances [4]. In response to this phenomenon, many companies (e.g., Alphabet, Amazon, Apple, and Microsoft) are investing in the development of mobile health applications designed to create an improved environment for monitoring individuals’ physical activity and personal health data, with the goal of enhancing both physical and mental well-being [5]. Consequently, mobile health applications have become an integral part of contemporary life, offering convenient tools to promote healthier lifestyles and improve healthcare access.

Despite their potential, the effectiveness of these applications heavily depends on the user experience they provide [6]. To further integrate these applications into daily routines, it is essential for users to perceive the various features as intuitive, user-friendly, and beneficial. Advanced features, if not accessible or intuitively designed, are unlikely to meet user expectations and may lead to dissatisfaction, thereby limiting their adoption and utility [7]. Moreover, given the increasing competition in the mobile health application market, companies face the challenge of not only developing advanced functionalities but also ensuring a seamless user experience that aligns with users’ diverse needs and preferences. This emphasizes the critical role of usability and perceived benefits in the sustained success of mobile health applications [8].

Understanding the factors that drive user engagement with mobile health applications can provide valuable insights into improving their design and functionality. Given this consideration, this study employed the Technology Acceptance Model (TAM) [9]. Because the TAM is based on the theory of reasoned action and the theory of planned behavior [10,11], this model is useful for understanding the process of how individuals perceive the function of technology and how these perceptions influence their future behavior [9]. Applying the features of the TAM, the purpose of this study is to examine the mechanism through which individuals perceive the ease of use and usefulness of the application, influencing their satisfaction and ultimately shaping their intention to accept the use of the application. By focusing on these aspects, this research seeks to bridge the gap between technological advancements and their effective integration into users’ daily healthcare practices. Because customers’ experience with products or services can provide a better way of advancing user-centered design, enhancing functionality, and improving overall satisfaction [12], we believe that this study serves as a stepping stone for organizations and developers to leverage insights to refine their offerings and drive long-term adoption.

### 1.1. Literature Review

#### 1.1.1. Technology Acceptance Model

The TAM illustrates how users adopt and interact with technology based on their perceptions of ease of use and usefulness, which, in turn, influence their attitudes and behavioral intentions [9]. Given this characteristic, the TAM has been applied in various contexts, including predicting user adoption, improving technology design, and enhancing business strategies [12,13,14]. In particular, the TAM has contributed to advancing knowledge of mobile applications by providing insights into how perceived ease of use and usefulness shape behavioral intentions. For example, Su and colleagues examined how user loyalty toward mobile food delivery applications develops through their experiences with the application and their perceptions of its functionality [15]. Similarly, in our context, mobile health applications offer various features for monitoring users’ health records and tracking their physical activities. However, there is limited understanding of how users evaluate different features within these applications, how their satisfaction is formed, and how these assessments influence their future behavior. To gain a deeper understanding of users’ evaluations of mobile health applications, it is essential to explore their perceptions of ease of use and usefulness, their satisfaction, and their behavioral intentions through the lens of the TAM.

#### 1.1.2. Perceived Ease of Use and Perceived Usefulness

Perceived ease of use refers to the extent to which individuals perceive that utilizing new technologies or systems requires minimal additional physical or cognitive effort [16]. Because the perception of ease of use can influence individuals’ adoption and utilization of the application [17], it is essential to present its functions in a clear and user-friendly manner. Especially since mobile health applications often include various functions that may overwhelm consumers, designing these applications to be user-friendly and for all ages is crucial for business success [18,19].

Perceived usefulness is associated with the degree to which individuals believe that using a specific system will lead to better performance in their job role [20]. Because perceived usefulness can reinforce individuals’ performance, it is acknowledged as a fundamental aspect of technology evaluation. Perceived usefulness has also been shown to exert both direct and indirect influences on attitudes toward technology use, thereby positively shaping the behavioral intention to adopt the technology [16,21,22]. For example, those involved in sport, including athletes and coaches, who acknowledge the value of an electronic competition scoring system are more likely to invest in it [23]. Therefore, perceived usefulness plays a crucial role in forming consumer attitudes and behaviors.

Furthermore, consumers’ views on the ease of using a product can shape their perception of the application’s overall usefulness. To enhance their perception of usefulness, it is crucial that applications are designed to ensure that their various features are readily accessible. For example, Schnall et al. found that the perceived ease of use of mobile health applications can positively influence users’ perception of the application [18]. Given this consideration, when users perceive the ease of use of mobile health applications, they are likely to recognize the usefulness of the application.

**H1.**  *Individuals’ perceived ease of use of mobile health applications will positively influence their perceived usefulness.*

#### 1.1.3. Consumer Satisfaction and Acceptance Intention

Customer satisfaction is a subjective evaluation or state of mind centered on the experience of goods, such as tangible and intangible goods as well as information [24]. It reflects a psychological response based on specific experiences and expectations [24]. Within the field of consumer behavior, researchers have extensively investigated predictors of consumer satisfaction across various contexts [25]. For example, fostering consumer satisfaction involves addressing their hedonic and utilitarian needs through social media applications [26]. For consumers to feel satisfied with using the applications, it is necessary to effectively represent the applications’ characteristics. However, previous research on mobile health applications has primarily focused on how their functionalities influence users’ overall evaluation of the applications and their behavioral intention to use them [27]. Because the evaluation of a specific brand is formed when consumers use the brand and experience satisfaction, it is essential to understand how individuals’ perceptions of ease of use and usefulness in mobile health applications influence their satisfaction with the applications.

**H2.**  *Individuals’ perceived ease of use of mobile health applications will positively influence their satisfaction with the applications.*

**H3.**  *Individuals’ perceived usefulness of mobile health applications will positively influence their satisfaction with the applications.*

Behavioral intention refers to consumers’ willingness or intention to continue adopting a particular product or service [28]. Because individuals’ behavioral intention is considered a precursor to actual use, it is important to understand how they respond to and engage with a product or service [28]. To emphasize the importance of behavioral intention, numerous studies have identified various predictors of behavioral intention, which are often used as a key outcome variable and highlighted as a critical factor in decision-making [29,30]. In this way, consumers’ behavioral intention to use a product or service is a significant factor in evaluating its functions and shaping their attitudes toward the product or service.

**Figure 1:**
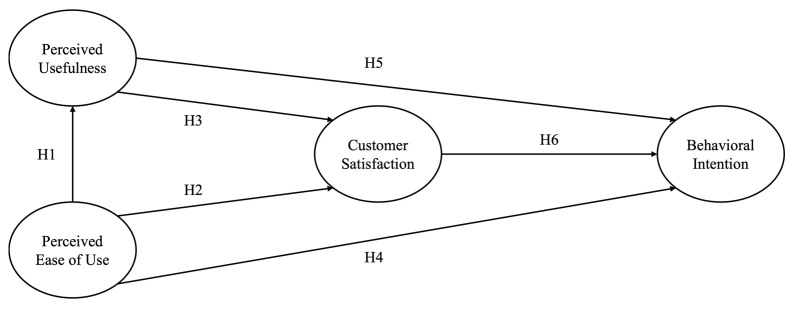
Research Model.

Several studies on mobile applications have highlighted that technological experience, usefulness, and attitudes are important in encouraging individuals’ behavioral intention [31,32]. For example, when individuals recognize the valuable advantages of the application, they want to use them in the future [31]. When users find an application intuitive and effortless to navigate, they are more likely to engage with it consistently and develop a positive attitude toward its use [12]. Users’ behavioral intentions are strengthened when they receive useful information from the application and positively evaluate their satisfaction with it [15]. Similarly, when mobile health applications are designed to be easy to use and provide valuable information, they encourage user engagement, foster positive experiences, and enhance perceived utility. As these experiences accumulate, consumers will assess whether to continue using the applications (See Figure 1).

**H4.**  *Individuals’ perceived ease of use of mobile health applications will positively influence their behavioral intention of the applications.*

**H5.**  *Individuals’ perceived usefulness of mobile health applications will positively influence their behavioral intention of the applications.*

**H6.**  *Individuals’ satisfaction with mobile health applications will positively influence their behavioral intention of the applications.*

## 2. Materials and Methods

### 2.1. Measurement

The mobile health applications utilized by research participants support a comprehensive range of well-being aspects, including physical activity, diet, sleep, and overall health management. This application enables automatic recording of daily activities and allows users to monitor various health metrics such as steps, workout intensity, heart rate, blood oxygen levels, and detailed sleep patterns, all enhanced by integration with wearable devices. Additionally, features such as goal-setting tools, flexible scheduling, and daily updates assist users in maintaining fitness habits and progressing at their own pace. To evaluate research participants’ perceptions and attitudes toward these mobile health applications, this study developed and utilized a survey questionnaire.

The questionnaire, which has demonstrated reliability and relevance, includes the following sections: (a) perceived usefulness, (b) perceived ease of use, (c) customer satisfaction, (d) behavioral intention, and (e) demographic information (e.g., gender, age, duration of application use, and average time spent on applications). Except for demographic information, all questionnaires were adapted on a five-point Likert-type scale ranging from 1 (*not at all*) to 5 (*very much*). Perceived usefulness and perceived ease of use were assessed using items from Venkatesh and Davis [21] and Agarwal and Karahanna [33], which demonstrated good reliability and validity. These items were adapted to fit the context of mobile health applications and to evaluate users’ experiences with these applications. Customer satisfaction was assessed using Oliver’s items [34] based on the context of mobile health applications. Behavioral intention toward using the applications was assessed using four items from Agarwal and Karahanna [33].

### 2.2. Procedures and Research Participants

**Figure 2:**
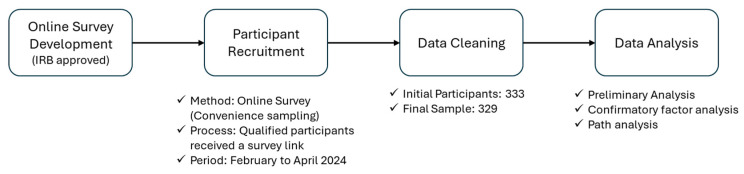
Flow chart of this research.

To better understand the complex research design and methods of this study, Figure 2 illustrates the major stages of the research process, outlining key information at each step (see Figure 2).

The target population of study was people who had experiences of using health mobile applications to see not only their perceptions but also attitudes for future behavior intentions. Participants were selected according to the following inclusion criteria: (a) 18 years of age or older, (b) residing in Korea, and (c) having experience using mobile health applications. To recruit potential participants, we utilized Macromill Embrain, which is one of the online recruitment companies in Korea. Participants who are qualified based on the criteria received an online survey link. Then, they answered with consent information before participating in the main survey. Of 333 participants, 4 were excluded for incomplete or rushed responses (e.g., survey completed in under two minutes instead of the expected five). Therefore, 329 final responses were analyzed. The survey was conducted from February to April 2024 and utilized a self-administered, read-and-fill-in questionnaire. Convenience sampling was employed among the non-probability sampling methods. The demographic characteristics of participants are shown in Table 1.

## 3. Results

The results section is structured to first present a preliminary analysis that evaluates representativeness of data and assess reliability and validity of variables. Following this, we conducted a confirmatory factor analysis (CFA) to evaluate the measurement model. The section then progresses to detailed path analysis, which examines the hypothesized relationships between perceived ease of use, perceived usefulness, customer satisfaction, and behavioral intentions regarding mobile health applications.

### 3.1. Preliminary Analysis

Before conducting structural equation modeling, data representativeness was evaluated in several steps using IBM SPSS version 29 and AMOS version 29. Composite Reliability (CR), Average Variance Extracted (AVE), Cronbach’s alpha coefficient, and latent variable correlations were examined to verify reliability and construct validity. The results of CR for all items ranged from 0.88 to 0.93, and the AVE for all items ranged from 0.64 to 0.74. The result of the Cronbach’s alpha coefficient showed above 0.80, indicating that there was no problem with internal consistency, which met the criteria proposed by [35,36]. Furthermore, the correlation results among variables in our study did not exceed 0.85 [37] and remained within the range of −1 to 1, supporting discriminant validity [38]. Thus, there were no multicollinearity or singularity issues, and our measurement model, including all constructs, was finalized (See Table 2).

The measurement was assessed through confirmatory factor analyses (CFA) to verify the validity and reliability of the survey produced using global fit indices: comparative fit index (CFI), Tucker–Lewis index (TLI), and Root Mean Square Error of Approximation (RMSEA). Our measurement model showed a good overall fit (*x*^2^ = 317.59, *df* = 121, CFI = 0.95, TLI = 0.94, RMSEA = 0.06), satisfying the criteria. Each item in this study was reviewed to check its factor loadings that reflect theoretical congruency and meaningfulness based on the suggestion (i.e., greater than 0.50 factor loading) [39]. The detailed results of CFA are shown in Table 3.

### 3.2. Path Analysis

With the acceptability of the measurement model, structural equation modeling was conducted to test the hypotheses. The structural model showed acceptable fit results (*x*^2^ = 304.53, *df* = 114, CFI = 0.96, TLI = 0.96, RMSEA = 0.07) (See Table 4).

**Figure 3:**
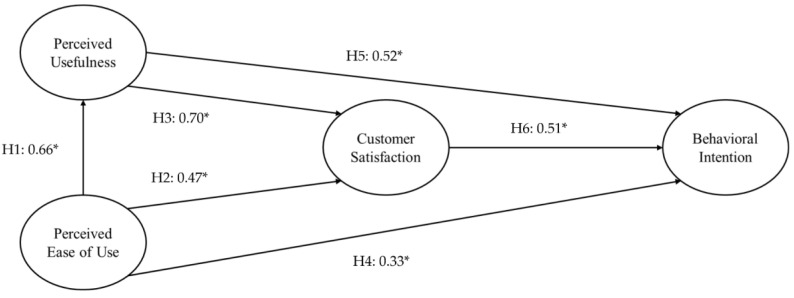
Hypotheses testing through structural equation modeling. * *p* < 0.001.

Figure 3 and Table 5 showed the results obtained upon confirming the suitability of the research model and verifying the causal relationship between variables. H1 explored the impact of the perceived ease of use of mobile health applications on the perceived usefulness of the applications, revealing a statistically significant path coefficient of 0.66 and a t-value of 9.43 (*p* < 0.001). Consequently, the hypothesis was confirmed and adopted, indicating a meaningful effect. H2 proposed that perceived ease of use of the applications influenced customer satisfaction with mobile health applications; the path coefficient of 0.47 and t-value of 6.11 established statistical significance (*p* < 0.001). Therefore, the hypothesis was confirmed and adopted, and there was a statistically significant effect. H3 examined the influence of the perceived usefulness of mobile health applications on customer satisfaction with the applications, revealing a significant path coefficient of 0.70 and t-value of 11.50 (*p* < 0.001). Statistical significance was confirmed, prompting the adoption of the hypothesis.

H4 investigated the influence of perceived ease of use of mobile health applications on behavioral intention to the applications, with a path coefficient of 0.33 and t-value of 3.61 (*p* < 0.001) found to be statistically significant. Regarding H5, which posited that the perceived usefulness of mobile health applications would influence behavioral intention to the applications, the path coefficient was found to be 0.52 and t-value was found to be 5.69 (*p* < 0.001), confirming statistical significance. H6, focusing on the influence of customer satisfaction with the applications on behavioral intention to mobile health applications, yielded a path coefficient of 0.51 and t-value of 5.13 (*p* < 0.001), with statistical significance confirmed. Hence, this hypothesis was accepted.

## 4. Discussion

The study explored the relationships among perceived usefulness, perceived ease of use, customer satisfaction, and behavioral intention to mobile health applications based on the TAM. We found empirical evidence supporting the role of perceived usefulness, ease of use, and satisfaction in shaping users’ behavioral intentions. Our first hypothesis proposed that perceived ease of use positively affects the perceived usefulness of mobile health applications. The result indicated a significant relationship between perceived ease of use and perceived usefulness, thereby supporting H1. This finding aligns with previous research, which suggests that the easier an application is to use, the more likely individuals are to continue using it consistently to perceive the usefulness of the applications [40]. Our finding provides more empirical evidence of the TAM, which highlights the role of perceived ease of access to technologies and tools to fulfill users’ expectations for efficiency.

The relationship between perceived ease and customer satisfaction with the applications was significant in the hypothesized direction, thus supporting H2. The users’ clear understanding of how to use the mobile health application enhances their satisfaction and overall positive experiences [41]. Thus, ease of use of mobile health applications is an imperative factor to positively influence users’ satisfaction. Additionally, the perceived usefulness of mobile health applications was found to positively influence customer satisfaction with the applications, thereby supporting H3. This finding aligns with previous research, especially for TAM, which suggests that individuals are more likely to be satisfied with mobile application services when these applications effectively address their daily challenges by providing valuable services or information, and users perceive tangible benefits from their use [42].

The findings of this research also confirmed the hypothesized relationship between perceived ease of use and behavioral intention to use mobile health applications (H4). This result is in line with prior research, which indicates that individuals who perceive banking applications as easy to use are more likely to continue using them in the future [12]. In this sense, when users perceive mobile health applications as easy to use, their intention to continue using the application is likely influenced by their overall perception of its usability and effectiveness. Furthermore, H5 was supported, as the finding revealed that the perceived usefulness of the applications positively influences users’ behavioral intentions. Previous research on perceived usefulness showed its significant effect on behavioral intention, highlighting its high reliability [8]. In this context, offering useful features in mobile health applications would encourage users to engage with and continue using the applications. H6 proposed that customer satisfaction with mobile health applications positively affects users’ behavioral intention toward these applications. The findings supported this hypothesis, showing that users with consistently satisfying experiences in utilizing services or benefits develop positive intentions to continue using the application. This satisfaction, built from specific experiences, has been shown to be a key driver of users’ future behavior [26]. Furthermore, customer satisfaction is recognized as a primary motivator for fostering not only acceptance but also long-term loyalty among users [43].

### 4.1. Theoretical Contribution and Practical Implication

This study offers several significant implications for both academics and practitioners. From a theoretical perspective, it broadens the application of the TAM to mobile health application services by empirically investigating the relationships between predictors and outcomes related to behavioral intention and usage within the context of the sport industry. Many studies on mobile applications have followed the TAM, though these studies have primarily focused on examining how the functions of an application influence users’ behavioral intentions [27,44,45]. In contrast, this research sheds light on users’ actual experiences, which shape their perception of the ease of use and usefulness of mobile health applications, ultimately influencing their satisfaction and, in turn, their behavioral intentions. Specifically, as users interact with mobile health applications, their hands-on experiences help them assess how intuitive and functional the applications are, reinforcing their perceptions of ease of use and usefulness. Positive experiences with seamless navigation and valuable features enhance user satisfaction, which subsequently strengthens their intention to continue using the application. This approach supports the previous research that highlights the significance of user experience in changing their health behavior [46].

Second, this study’s contribution is to identify the influence of mobile health application services on encouraging consumers’ behavioral intentions. Previous mobile application services studies in the sport industry have focused on reinforcing the relationship between sport consumers and their favorite team [45,47] and improving their motivation for the team’s content [48]. Departing from studies centered on applications for sport consumers, our research focused on individuals who use mobile health applications are influenced in their behavioral intention to use the applications. Specifically, it examined how users perceive the usability of these application services, their attitudes toward the applications, and how these perceptions of functionality and satisfaction shape their behavioral intentions.

The findings of our research provide practitioners with valuable insights into developing and implementing mobile health application services, as well as designing effective marketing strategies for their organizations. First, the mobile application industry has the potential to expand its market share due to advancements in mobile health application technology. If these application services can offer tailored workout suggestions based on users’ accumulated data, customers are likely to develop positive evaluations of this functionality. In the current landscape, consumers increasingly recognize the unique value of personalized activities, which may foster loyalty to the application [49]. Leveraging these positive evaluations, practitioners can strategically promote their services to gyms or community groups where individuals engage in sports activities. Consequently, businesses and organizations within these sectors can anticipate increased profitability.

Second, our findings are expected to help sports organizations gain a deeper understanding of how individuals perceive the usefulness and ease of use of mobile health application services. This enhanced understanding will enable organizations to effectively demonstrate the applications’ utility and ease of use to users, thereby accelerating the development of services integrated with these applications. For instance, the application could include a comparison feature that visualizes users’ progress before and after following specific fitness routines. It might also provide weekly health assessments and tips for improvement based on user data. These features not only make the app easy to use but also highlight its utility, encouraging greater adoption among fitness enthusiasts. As a result, the sport organization could promote this application as part of its community engagement initiatives, driving brand loyalty and additional revenue streams through subscriptions or sponsorships.

### 4.2. Limitation and Future Research

Although this research study yields meaningful outcomes, it is not without limitations. First, this research employed convenience sampling, which may not accurately represent potential users of mobile health applications [50]. Future researchers would benefit from utilizing proportional stratified random sampling to more effectively understand the perceptions and preferences of each target group. Furthermore, it is also important to consider participants’ age and gender. Prior research on information systems and information and communication technology suggests that age and gender differences significantly influence perceptions and behaviors. For example, younger individuals tend to use near-field communication mobile payments more frequently than older individuals due to their lower perception of the technology’s risks [51]. Additionally, women are more likely to adopt this technology when they are satisfied with the payment system compared to men. Given these considerations, future studies should adopt a more targeted approach, examining specific age cohorts or generational groups to better capture these nuances.

Second, the current research investigated the effects of mobile health applications using quantitative methods. To enhance the general understanding gained through surveys, future research should consider employing qualitative methodologies to gain deeper insights into user experiences. This approach would facilitate a comprehensive exploration of participants’ satisfaction, challenges, and attitudes toward their engagement with mobile health applications.

## 5. Conclusions

This study aims to enhance understanding of mobile health applications in the sport industry and the broader mobile health sector by examining a model that evaluates users’ perceptions of application services and identifies key predictors of satisfaction and behavioral intentions. The findings confirm that perceived usefulness and perceived ease of use are significant predictors of customer satisfaction, while behavioral intentions are shaped by perceived usefulness, ease of use, and satisfaction. As an initial empirical investigation, this research provides a comprehensive and integrative analysis, offering valuable insights into the pivotal role of mobile health applications in both the sport industry and the wider mobile health market.

## Figures and Tables

**Table 1:** Demographic characteristics of participants.

Characteristics	Classification	Frequency (n)	Percentage (%)
Gender	Male	165	50.2
	Female	164	49.8
Age	20’s	70	21.3
	30’s	66	20.1
	40’s	66	20.1
	50’s	65	19.8
	Over 60’s	62	18.7
Duration of Application Use	less than 1 year	28	8.5
	1–2 years	29	8.8
	2–3 years	74	22.5
	3–4 years	81	24.6
	more than 4 years	117	35.6
Average Time Spent on Application	less than 1 h	175	53.2
	1–2 h	88	26.8
	2–3 h	39	11.9
	3–4 h	17	5.2
	more than 4 h	10	2.9
Total		329	100

**Table 2:** Correlation Analysis.

	1	2	3	4
1. Perceived Usefulness	1			
2. Perceived Ease of Use	0.67 *	1		
3. Customer Satisfaction	0.65 *	0.68 *	1	
4. Behavioral Intention	0.63 *	0.65 *	0.56 *	1

* *p* < 0.01.

**Table 3:** Results of CFA for the measurement model.

Factors	Items	Estimate	S.E.	CR	AVE	α
Perceived Usefulness	Using the mobile health application enhances my health.	0.88	0.24	0.91	0.74	0.91
	Using the mobile health application increases my physical activity level.	0.82	0.35			
	Using the mobile health application provides useful information.	0.85	0.26			
	I find the mobile health application provides interesting information.	0.88	0.28			
Perceived Ease of Use	Learning to operate the Web is easy for me.	0.86	0.16	0.88	0.64	0.89
	I find it easy to get the mobile health application to do what I want it to do.	0.80	0.24			
	It is easy for me to become skillful at using the mobile health application.	0.76	0.25			
	I find the mobile health application easy to use.	0.78	0.23			
Customer Satisfaction	Overall satisfied with using mobile health applications for healthcare.	0.85	0.30	0.93	0.73	0.93
	Healthcare through mobile health applications is a smart thing to do.	0.91	0.21			
	Healthcare through mobile health applications meets my expectations.	0.83	0.37			
	Healthcare through mobile health applications is the right decision.	0.83	0.35			
	I am satisfied with my decision to use mobile health applications for healthcare.	0.84	0.31			
Behavioral Intention	I intend to use mobile health applications in the future.	0.79	0.17	0.90	0.68	0.89
	I will use mobile health applications in the future.	0.82	0.24			
	I will talk positively about mobile health applications to others.	0.84	0.29			
	I will recommend using mobile health applications to others.	0.85	0.16			

**Table 4:** Model fit of hypothesized model.

ꭓ^2^	*df*	TLI	CFI	RMSEA
304.53	114	0.96	0.96	0.07

**Table 5:** Results of path analysis and hypothesis verification.

H	Pathway	Path Coefficient	S.E.	t	Accept /Reject
H1	Perceived Ease of Use → Perceived Usefulness	0.66	0.05	9.43 *	Accept
H2	Perceived Ease of Use → Customer Satisfaction	0.47	0.07	6.11 *	Accept
H3	Perceived Usefulness → Customer Satisfaction	0.70	0.07	11.50 *	Accept
H4	Perceived Ease of Use → Behavioral Intention	0.33	0.07	3.61 *	Accept
H5	Perceived Usefulness → Behavioral Intention	0.52	0.07	5.69 *	Accept
H6	Customer Satisfaction → Behavioral Intention	0.51	0.10	5.13 *	Accept

* *p* < 0.001.

## Data Availability

The data presented in this study are available on request from the corresponding author.

## Acronyms

CFA	Confirmatory factor analysis
CFI	Comparative Fit Index
CR	Composite reliability
SEM	Structural Equation Modeling
TAM	Technology Acceptance Model
TLI	Tucker-Lewis Index
RMSEA	Root Mean Square Error of Approximation
